# Rapid and simultaneous multiepitope antigen-based detection of *Enterococcus* by microscale thermophoresis and immunomagnetic separation

**DOI:** 10.3389/fmicb.2024.1341451

**Published:** 2024-01-23

**Authors:** Yan Liu, Ziyan Wang, Ze Wang, Jun Zhou, Jiaojiao Han, Chenyang Lu, Bing Liu, Rongxian Yu, Xiaoling Sun, Zhen Zhang, Rixin Wang, Xiurong Su

**Affiliations:** ^1^State Key Laboratory for Quality and Safety of Agro-products, Ningbo University, Ningbo, China; ^2^School of Marine Science, Ningbo University, Ningbo, China; ^3^Vigor Health Products Co., Ltd., Shenzhen, China

**Keywords:** *Enterococcus*, multi-epitope antigens, dltD, microscale thermophoresis, pathogen detection

## Abstract

**Background:**

Generally, enterococci bacteria cause nosocomial infections and are major indicators of bacterial contamination in marine bathing beach. However, a method for the rapid and simultaneous detection of multiple pathogenic enterococci has not been developed on account of the wide variety of pathogenic enterococci and their existence in complex matrices.

**Methods:**

Immunoinformatics tools were used to design a multi-epitope antigen for the detection of various pathogenic enterococci by using the sequence of dltD gene on enterococci lipoteichoic acid (LTA) surface, which is associated with toxicological effects. The multi-epitopes included enterococci such as *Enterococcus faecalis*, *E. gallinarum*, *E. raffinosus*, *E. durans*, *E. faecium*, *E. hirae*, *E. thailandicus*, *E. casseliflavus*, *E. avium*, *E. mundtii*, *E. lactis*, *E. solitarius*, *E. pseudoavium*, and *E. malodoratum*. Microscale thermophoresis (MST) and western blot were carried out to detect the affinity between multi-epitope antigens and antibodies and between multi-epitope antibodies and bacteria. Furthermore, the detection of pathogenic enterococci was carried out by using immunomagnetic beads (IMBs) and immune chromatographic test strip (ICTS).

**Results:**

The multi-epitope antibody had a satisfactory affinity to the antigen and enterococci. IMBs and ICTS were detected with a minimum of 101 CFU/mL and showed incompatibility for *Vibrio parahemolyticus*, *V. vulnifcus*, *V. harveyi*, *V. anguillarum*, and *Edwardsiella tarda*.

**Implication:**

The present study demonstrated that the multi-epitope antigens exhibited excellent specificity and sensitivity, making them highly suitable for efficient on-site screening of enterococci bacteria in marine bathing beaches.

## 1 Introduction

As Chinese economy by leaps and bounds, people’s living standard is constantly increasing, thus increasing the demand for tourism recreation. Considering the unique natural resources and broad development prospects, coastal tourism is considered to be the focus of development. As an important part of coastal tourism resources, the bathing beach provides tourists with places of relaxation and leisure and promotes national economic development (Inácio et al., 2022). However, some bathing beach areas do not meet quality standards according to EU reports (Agency, 2019). The health risks of beachgoers are related to exposure to beach water, and polluted seawater poses a threat to human health (Dufour, 2021). According to a 2001 WHO report, the majority of bathing beach illnesses are gastrointestinal, but this condition can be reduced by assessing the microbiota of beach water. Currently, the microbial parameters used in beach water quality assessment can be divided into three types, namely, total *Escherichia coli*, fecal *E. coli*, and enterococci (Hsu and Huang, 2008). Pursuant to the plan of Action Plan for Beaches and Recreational Water published by the USEPA in 1999, enterococci are the primary indicator used to assess the bacterial contamination of bathing water, and for marine water quality testing, enterococcal indicators are more applicable than total *E. coli*. Furthermore, the measurement method based on enterococci is effective in monitoring beach recreational waters, because diseases in marine recreational waters are mainly caused by enterococci (Wade et al., 2008, 2010).

*Enterococcus* is a Gram-positive bacteria that is ubiquitous microorganism in nature, and it exists mainly in the feces of humans and other animals in the form of symbionts, and the number of its flora is second only to *E. coli* (Yerlikaya and Akbulut, 2019). In addition, enterococci are important opportunistic pathogens and have been recognized worldwide as the leading cause of infection and one of the most common nosocomial pathogens (Fiore et al., 2019). Combined with the misuse of antibiotics in recent years, their rapid epidemic has also created alarming resistance, thus increasing the risk of treatment failure and death in infected people (García-Solache and Rice, 2019; Miller et al., 2020). Besides *Enterococcus faecalis* and *E. faecium*, which mainly cause human diseases, several kinds of enterococci can also cause disease infections, including *E. durans*, *E. mundtii*, *E. casseliflavus*, *E. hirae*, and *E. lactis* (Bollam et al., 2021). It can cause bacteremia, urinary tract infection, endocarditis, peritonitis, and arthritis (Okumura et al., 2021; Oldberg and Rasmussen, 2021). The use of qPCR has been proposed for enterococcus detection to determine beach water quality. The method was refined and applied to the water quality testing of seawater and freshwater resources by EPA’s National Environmental and Epidemiological Evaluation Study (NEEAR) (Dufour, 2021). As a result, by detecting the number of enterococci in the environmental source in advance, the water quality detection of the bathing beach area can be realized, which can effectively avoid the pollution of the water environment and reduce the possibility of disease. DltD is a membrane protein that is mainly found in Gram-positive bacteria and acts directly on lipoteichoic acid (LTA) acylation (Wood et al., 2018). LTA, a surface polymer of Gram-positive bacteria, is an important surface antigen that is involved in host–microbe interactions, as well as in maintaining bacterial membrane homeostasis and virulence, and it is one of the main pathogenic components of bacteria (Wei et al., 2021). DltD plays an important role in antigen–antibody interactions (Wood et al., 2018).

Wienken et al. (2010) used MST technology to measure protein–protein interactions in solution, which has aroused great interest among researchers. It only requires trace amount of target protein labeling solution mixed with ligand to perform on-machine detection. It is often used in the early detection of the affinity of two substances. Manna et al. (2022) used MST to directly assess the ability of a cyclic peptidomimetic of kinase inhibitory region-suppressors of cytokine signaling 1 (KIR-SOCS1), icPS5 (Nal1), to recognize the catalytic domain of Janus kinase 2 (JAK2), which provides additional possibilities for the treatment of neurological, autoimmune, or cardiovascular diseases. The detection of pathogenic microorganisms mainly includes the pre-treatment of samples and the detection of microorganisms (Lv et al., 2021). The traditional pretreatment methods of microorganisms are membrane filtration and centrifugation. Although these methods entail low cost, the filters are easily clogged by the matrix and lack selectivity, and repeated separation and washing reduce the recovery rate, causing wastage of samples and loss of bacteria (Garrido-Maestu et al., 2018; Pan et al., 2020). The main methods used for pathogen detection mainly include quantitative reverse transcription-polymerase chain reaction (RT-qPCR), polymerase chain reaction (PCR), immune chromatographic test strip (ICTS), droplet digital PCR (DDPCR) and recombinase polymerase amplification (RPA) (Cui et al., 2022). Considering that the content of target microorganisms is low, and the samples often contain many non-target bacteria and other impurities, which affect the detection results and efficiency, the detection technology must be combined with effective sample pre-treatment (Wang et al., 2020). Nowadays, magnetic beads (MBs) based on magnetic solid phase extraction has attracted significant attention because of their advantages such as specific surface area and simple separation process. By using this advantage, immunomagnetic separation (IMS) has successfully pretreated and concentrated bacteria to eliminate inhibitors in the sample matrix, thus shortening the enrichment time. It has been used in combination with various detection methods for pathogen detection. IMS combined with LAMP can be used for the rapid detection of *Salmonella* in duck meat (Bi et al., 2020), combined with PCR for detection of *Cronobacter* spp. (Chen et al., 2017). Nevertheless, these analytical methods require complex operations and specialized laboratories, making them unsuitable for *in situ* and visible testing, especially in some tourist and leisure areas (Esteve-Turrillas et al., 2018). By contrast, ICTS technology enables fast and reliable *in situ* detection (Lei et al., 2020), and compared with the traditional serological analysis method, this method has high sensitivity and low requirements on detection environment and equipment and can be used as a rapid and sensitive screening method for pathogenic bacteria (Nagasawa et al., 2020). Moreover, the simultaneous detection of multiple pathogenic enterococci by using the IMS-ICTS assay has not been reported.

In the study, a novel multi-epitope antigen with high sensitivity and specificity has been proposed for the rapid trace detection of enterococci microscopically. The antigen was composed of the antigenic sequences of enterococci, and multi-epitope antibodies were obtained by immunizing mice. MST was used to detect the affinity of bacteria and antibodies *in vitro*, and IMS and ICTS techniques were used for enterococcus detection. The prepared synthetic multi-epitope antibodies were systematically evaluated with the relevant experimental results. This study aims to bring novel ideas and methods for the rapid pathogen detection of enterococci in marine bathing beach and to promote the development of coastal tourism industry and provide a scientific basis for building a world-class bathing beach.

## 2 Materials and methods

### 2.1 Bacterial strains and culture conditions

The bacteria used in this study, including *Enterococcus durans* (Ed), *E. mundtii* (Enm), *E. casseliflavus* (Ec), *E. hirae* (Eh), *E. lactis* (Enl), *E. faecalis* (Ef), *E. faecium* (Enf), *Vibrio parahemolyticus* (Vp), *V. vulnifcus* (Vv), *V. harveyi* (Vh), *V. anguillarum* (Va), and *Edwardsiella tarda* (Et) were obtained from the laboratory, and then cultured in Brian heart infusion (BHI), each 1 L medium contained 10.0 g peptone, 12.5 g dehydrated calf brain infusion 5.0 g, dehydrated buffalo heart infusion 5.0 g, sodium chloride 5.0 g, glucose 2.0 g and 2.5 g disodium hydrogen phosphate (pH 7.4 ± 0.2) and beef extract-peptone medium (each 1 L medium contained 3 g beef extract, 10 g peptone, 5 g NaCl and 15–20 g agar, pH 7.4–7.6) overnight at 28°C in a rotary shaker before use.

### 2.2 Preparation of multi-epitope antigens and antibodies

This experiment identified the amino acid sequences of epitopes on dltD of 14 common enterococcal lipoteichoic acid (LTA) proteins by using bioinformatics method. The dltD sequences of 14 enterococci were extracted from the National Coalition Building Institute (NCBI^1^), while based on the principle of similarity, sequence alignment was carried out. The epitope prediction tool, namely, Immune Epitope Database (IEDB^2^) was used to analyze and predict the epitope exposed outside of dltD. The amino acid fragments of the epitopes were screened out according to the score and map, and then the epitopes were connected by flexible peptide connectors (GGGGS) The order of each epitope was rearranged and optimized, and then analyzed again by using the multi-epitope tool. The optimal sequence of epitopes was selected, and a His-tag for purification was added to the end of the epitope, which was named En dltD. The 3D structure prediction tool^3^ was used to predict its structure and synthesize multi-epitope antigen artificially. To produce En dltD, the gene was inserted into pET-28a expression vector (Novagen, Germany) (termed pET-28a-En dltD). The recombinant plasmids were transformed into *E. coli* Rosetta (DE3) (Novagen, Germany) and subjected to DNA sequencing. The positive clones were subsequently incubated in LB medium and induced expression of En dltD. Plasmids containing En dltD were transformed into plasmid-expressing competent cells, and the expression of proteins was induced, and the protein was purified using Ni-NTA nickel ion affinity chromatography column (GE Healthcare, United States) chromatography. The molecular weight and purity of elution peak were analyzed by 12% SDS-PAGE, and the concentration of multiple epitope antigen was determined using the BCA protein concentration determination kit from Kangwei Century Biology Co., Ltd. (Jiangsu, China). BALB/c male mice were immunized by intraperitoneal injection with an interval of 7 days. The reagents used were antigens mixed with complete and incomplete Freund’s adjuvant. After the fourth immunization, the mice were fasted for 24 h, and blood samples were collected from the tails after administration of anesthesia to obtain serum containing artificial polyepitope antibodies. The sample was stored at −80°C.

### 2.3 Separate detection of the interaction of antibody and antigen and bacteria

#### 2.3.1 Western bolt verification of En dltD and multi-epitope antibodies

The BCA protein detection kit from Kangwei Century Biology Co., Ltd. (Jiangsu, China) was used for En dltD concentration determination. The sample was then analyzed using 12% SDS-PAGE and transferred to PVDF membrane (Merck, United States), and the membrane was sealed with 5% skim milk powder, and the multi-epitope antibodies were incubated overnight at 4°C as primary antibodies, and then with the secondary antibody bound with HRP. Antibodies and skim milk powder were obtained from Shanghai Sangon Biotech Co., Ltd. (Shanghai, China). The final results were observed using a gel imager (Bio-Rad, United States).

#### 2.3.2 Qualitative analysis and detection by MST technology

Seven representative and more common enterococci (Ec, Ef, Enl, Eh, Enf, Enm, and Ed) were selected to complete the following detection. The Monolith^™^ RED-NHS second-generation protein labeling kit was used. The NHS-ester carried by RED dye can be covalently bound to the primary amino group (lysine residue), which is suitable for Monolith^™^ NT.115 series and NT. Automated series instruments were equipped with red light detectors (Nano and Pico). According to the kit instructions, multi-epitope antibodies were labeled with fluorescent molecules as receptors. Enterococci were used as ligands. The affinity test can only be carried out after the fluorescence range is between 200 and 2,000, and the classic curve of MST has appeared. The labeled receptors were mixed at 1:1 ratio with PBS and ligand solution in parallel for four times. Finally, the test and experimental results were analyzed. Notably, the adsorption of the fluorescent target to capillary walls will cause irregularities in the shape of capillary peaks in the capillary scan. It immobilizes the target and should therefore be avoided, and the detection results are meaningful only if the samples are free of aggregation, and the fluorescence fluctuation range is within 20%. Standard capillaries were employed for analysis.

### 2.4 Nano-immunomagnetic bead analytical technique

#### 2.4.1 Preparation of nano IMBs

Nano Fe_3_O_4_ was re-suspended, and 400 μL of sample was transferred to a new 1.5 mL centrifuge tube. After magnetic separation, the supernatant was removed, 1 mL of 15 mM morph methanesulfonic acid (MES pH 6.0) buffer was added, and the sample was shaken for 5–10 s. Then, the centrifugal tube was placed on the magnetic rack for magnetic separation for 3 min, the supernatant was removed, and the process repeated for 2–3 times. The beads were re-dispersed in 100 μL of 15 mM MES (pH 6.0) buffer with 100 μL of EDC. After incubation at room temperature for 30 min, the centrifuge tube was placed on a magnetic rack for magnetic separation for 3 min, and the supernatant was removed. Afterward, 50, 100, 200, 400, and 800 μg multi-epitope antibodies were added to magnetic beads and diluted to 200–500 μL with 15 mM MES (pH 6.0) buffer, and then incubated overnight at room temperature. The centrifugal tube was placed on the magnetic rack for magnetic separation for 3 min to remove supernatant, 1 mL of PBS (containing 0.1% Tween-20) was added, and the centrifugal tube was placed on the drum for mixing for 10 min. After magnetic separation the supernatant was removed, and the process was repeated. Approximately 1 mL of PBS (containing 0.1% tween-20 and 0.1% BSA) was added into each tube to re-disperse the IMBs, and the sample was stored at 4°C.

#### 2.4.2 Optimization of nano IMBs

The trapping ability of five kinds of MBs to different enterococci was studied (50, 100, 200, 400, and 800 μg/mL) with four IMB dosages (0.75, 1.5, 3, 6, and 12 mg), six immunoreaction times (5, 15, 30, 45, 60, and 90 min), five magnetic separation times (1, 3, 5, 7, and 9 min), and four different buffer pH values (pH 6.0, 7.4, 8.0, and 9.6) (Lv et al., 2021).

Enterococci were diluted with sterile PBS after inoculated in BHI (10 mL) overnight at 28°C with sharking and diluted with sterile PBS. Five 1.5 mL centrifuge tubes containing 3 mg of IMB samples with 50, 100, 200, 400, and 800 μg/mL multi-epitope antibodies were added with 1 mL of enterococci suspension. A blank control without IMBs was also prepared. All centrifuge tubes were placed on a tube rotator for 45 min at 37°C. After magnetic separation for 3 min, and the liquid phase was transformed to another centrifuge tubes, and the sediment was washed thrice with PBS. Finally, the residual bacteria compounds were re-dispersed in 1 mL of PBS and inoculated on 3 M petrifilm plates with three parallel tests. The 3 M petrifilm plates were performed at 28°C for 1–2 days, and the capture efficiency (CE) was calculated using the following equation (Bi et al., 2020): CE (%) = (*N_0_* – *N_1_*)/*N_0_* × 100%, where *N_0_* and *N_1_* are the initial and residual numbers of bacteria in the samples, respectively. The optimization of the other four capture conditions of the IMBs was carried out the same as above. Under the optimal capture conditions, the microscopic structures of the IMBs and bacteria were observed. IMBs and enterococci were combined in PBS. After fixation with glutaraldehyde and dehydration by gradient ethanol, the combination of IMBs and enterococci was observed under scanning electron microscopy (SEM).

#### 2.4.3 Application of IMBs in the detection of *Enterococcus*

IMBs were mixed with 1 mL of enterococci suspensions of different species, and the range of bacterial concentration was 10^1^–10^5^ CFU/ mL. The samples were mixed by rotation at 37°C for 45 min magnetic separation, washed thrice with sterile PBS, resuspended with PBS, and inoculated on 3 M petrifilm plates with three parallel samples. The samples were cultured at 28°C for 1–2 days, and the capture efficiency and minimum capture limit were calculated. Likewise, Vp, Vv, Vh, Va, and Et values of 10^5^ CFU/mL were selected to complete the specific detection of IMBs. Thereby, the disturbance resistance of the IMBs was determined.

### 2.5 Detection by immune colloidal gold

#### 2.5.1 Synthesis of the immune colloidal gold

Colloidal gold was prepared by trisodium citrate reduction (Liu et al., 2020; Wangman et al., 2020). The quality of immune colloidal gold was determined by transmission electron microscopy (TEM) and using a UV–visible scanning spectrophotometer. At the same time, observe colloidal gold solution is clear, no suspended matter and precipitation, in order to judge the quality of colloidal gold.

Magneons and 30 mL of colloidal gold solution was placed into the conical flask. The mixture was stirred slowly on a magnetic stirrer. K_2_CO_3_ solution (0.1 mol/L) was added to adjust the pH value, then the best labeled amount of antibody was added slowly, and the mixture was stirred slowly for 2 h. Then, the 1/10 volume of 10% bovine serum albumin (BSA) of PBS was added to the colloidal gold conjugation and incubated for 30 min to bind colloidal gold residue sites. The mixture was centrifuged at 3,000 rpm for 5 min at 4°C, the precipitate was removed, the supernatant was centrifuged at 10,000 rpm for 30 min at 4°C (Wu et al., 2021). Finally, the precipitate was dispersed in in buffer solution containing 1% (*m*/*v*) sucrose, 0.5% (*v*/*v*) Tween-20, and 1% (*m*/*v*) BSA, the resulting solution is 1/10 the volume of the original colloidal gold solution and the sample was stored at 4°C for further use. The detail of gold label protein optimizing methods is available in Supplementary materials and methods.

#### 2.5.2 ICTS structure and performance testing.

The multi-epitope antibody and goat anti-mouse IgG were dispensed as test (T) and control lines (C), respectively. The dot film machine was applied to the upper and lower parts of the NC membrane. The NC membrane (300 mm × 25 mm) with two dispensed lines (the distance between the T line and C line is 0.5 cm, 1 μL/cm) was pasted on the center of the bottom plate. The NC film was dried in an oven at 45°C for 1 h, and the sample pad and joint point were pretreated with buffer and then dried in 45°C for 1 h. Then, the immune colloidal gold solution was evenly poured onto the glass fiber membrane and oven-dried at 45°C for 1 h. Then. the processed gold standard pad, NC film, sample pad. and absorbent pad were glued to the PVC bottom plate. The assembly was cut into 4 mm-wide strips for usage.

Enterococci were diluted in 10-fold gradient with sterile PBS to a concentration of 10^1^–10^5^ CFU/mL. The five concentrations of enterococci liquid were added to the colloid gold test strip adding sample wells, and the sensitivity test was carried out with PBS was used as negative control. In the absence of color at T and in the presence of one band at the C, the results were determined to be negative. By contrast, in the presence of strips on both lines C and T, the result was evaluated as positive. If only the T line has a strip or wireless is present, the result was considered invalid.

### 2.6 Detection in pure culture samples of *Enterococcus* of IMS-ICTS

In the previous experiments, IMBs solved the problems of easy loss of trace samples and substrate inhibition in pretreatment. Rapid trace detection was achieved by combining IMBS with ICTS. Ten 1.5 mL EP tubes were divided into two groups. One group was supplemented with Ef and IMBs at a concentration of 10^1^–10^5^ CFU/mL, and the other one was only supplemented with Ef at a concentration of 10^1^–10^5^ CFU/mL. This group was used as negative control, the reaction was carried out on a rotary mixer at 37°C for 45 min, and then resuspended in 100 μL of PBS after magnetic separation. The resuspended solution was placed in a metal bath at 100°C for 15 min, followed by magnetic separation. ICTS was used to detect the supernatant obtained from the two groups of experiments, and the supernatant was waited for 10 min. The results were evaluated according to the color changes of C and T line in ICTS. Performance test of other enterococci was carried out using the method above.

## 3 Results and analysis

### 3.1 Preparation of artificial multi-epitope antigen and antibody

**Figure 1:**
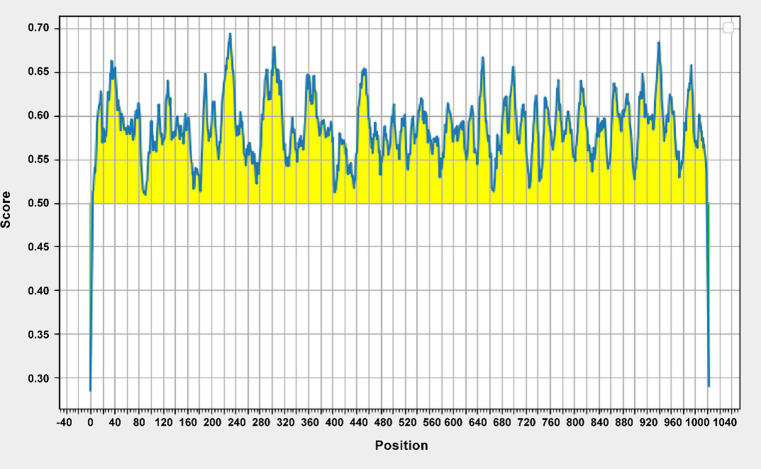
Epitope sequence of 14 enterococcal proteins obtained by recombination after evaluation conservative region of En dltD epitope. The 14 types of enterococci include Ef, Eg, Er, Ed, Enf, Eh, Ent, Ec, Ea, En, Enl, Es, Ep, and Em.

**Figure 2:**
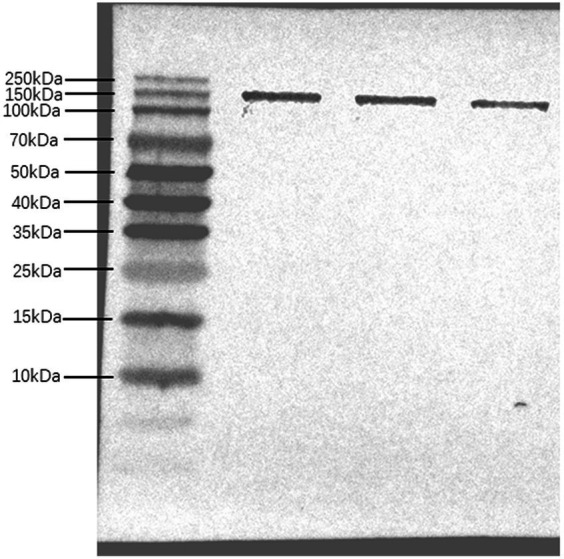
Western Bolt analysis of multi-epitope antibody. Multi-epitope antigen En dltD was isolated by SDS-PAGE and analyzed by western blot, and the three bands in western blot analysis were parallel.

The epitope prediction tool was used to screen the dominant epitope sites for multi-epitope fusion expression (Figure 1). Flexible peptides (GGGGS) were used to link different epitope sites. After further optimization, a His-tag was added to the N-terminus to obtain the multi-epitope antigen sequence En dltD. The multi-epitope antigen En dltD has a molecular weight of approximately 120 kDa and has good antigenicity. Samples were retained at each step of protein expression, and the protein bands were identified by 12% SDS-PAGE analysis between 100 and 140 kDa with molecular weights, which were very close to the predicted theoretical molecular weights (Supplementary Figure S1). Western blot analysis of serum from immunized mice showed that the serum produced by the constructed multi-epitope antigen can generate specific bands with En dltD (Figure 2), confirming that the polyclonal antibody had decent reactogenicity with the constructed polyepitope antigen.

### 3.2 MST technology to detect antibody affinity

**Figure 3:**
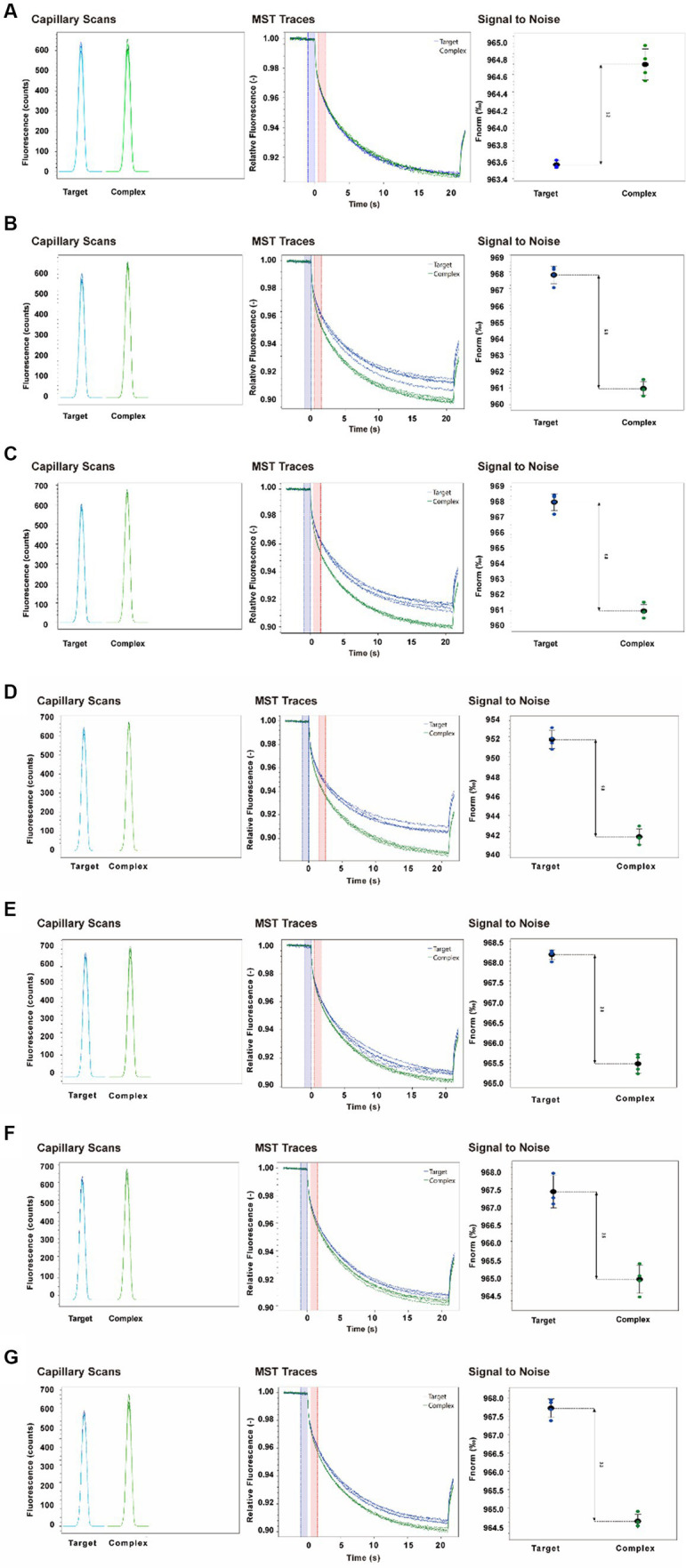
Affinity results of multi-epitope antibody detected by MST technique. The results were the affinity detection of **(A)** Ec, **(B)** Ef, **(C)** Enl, **(D)** Eh, **(E)** Enf, **(F)** Enm, and **(G)** Ed with antibodies, the target is the marker protein and buffer, complex is the marker protein and ligand, and each group has four parallels.

Binding Check program in MO was employed. Control software was used to detect the affinity of multiple epitope antibodies and seven enterococci. The Capillary Scans provides information about the overall fluorescence intensity and molecule adsorption to capillary walls for each individual capillary. The MST Traces overview provides feedback about the aggregation of the target molecule and photobleaching properties of the samples. It shows the reflection of the MST signal of the fluorescent target molecule and complex. The Signal-to-Noise ratio was used to evaluate the quality of the binding data. It is defined as the response amplitude divided by the noise of the measurement. A signal-to-noise ratio of more than 5 is desirable. The results of Capillary Scans and MST Traces of enterococci showed that the shape of capillary peak was regular without abnormal fluctuation. Then, the fluorescence of labeled proteins was in the range of 200–2000, and the fluorescence values of parallel samples were stable and fluctuated within 20%. The sample in the capillary tube was irradiated with the infrared laser from the inside of the instrument, causing the micro temperature gradient, thermal swimming phenomenon, and the typical MST curve (Figures 3A–G). After passing the first two steps, the Signal-to-Noise results showed that the signal-to-noise ratio of enterococci was more than 5, showing that the multi-epitope antibody could specifically recognize and bind seven enterococci. The signal-to-noise ratios of Ec, Ef, Enl, Eh, Enf, Enm, and Ed were 10.3, 14.4, 17.7, 11.5, 15.4, 5.8, and 14.1, respectively. All the seven enterococci had affinity with polyepitope antibodies, thus providing a possibility for subsequent experiments.

### 3.3 Condition optimization for bacterial capture with IMBs

#### 3.3.1 Optimization of antibody dosage

**Figure 4:**
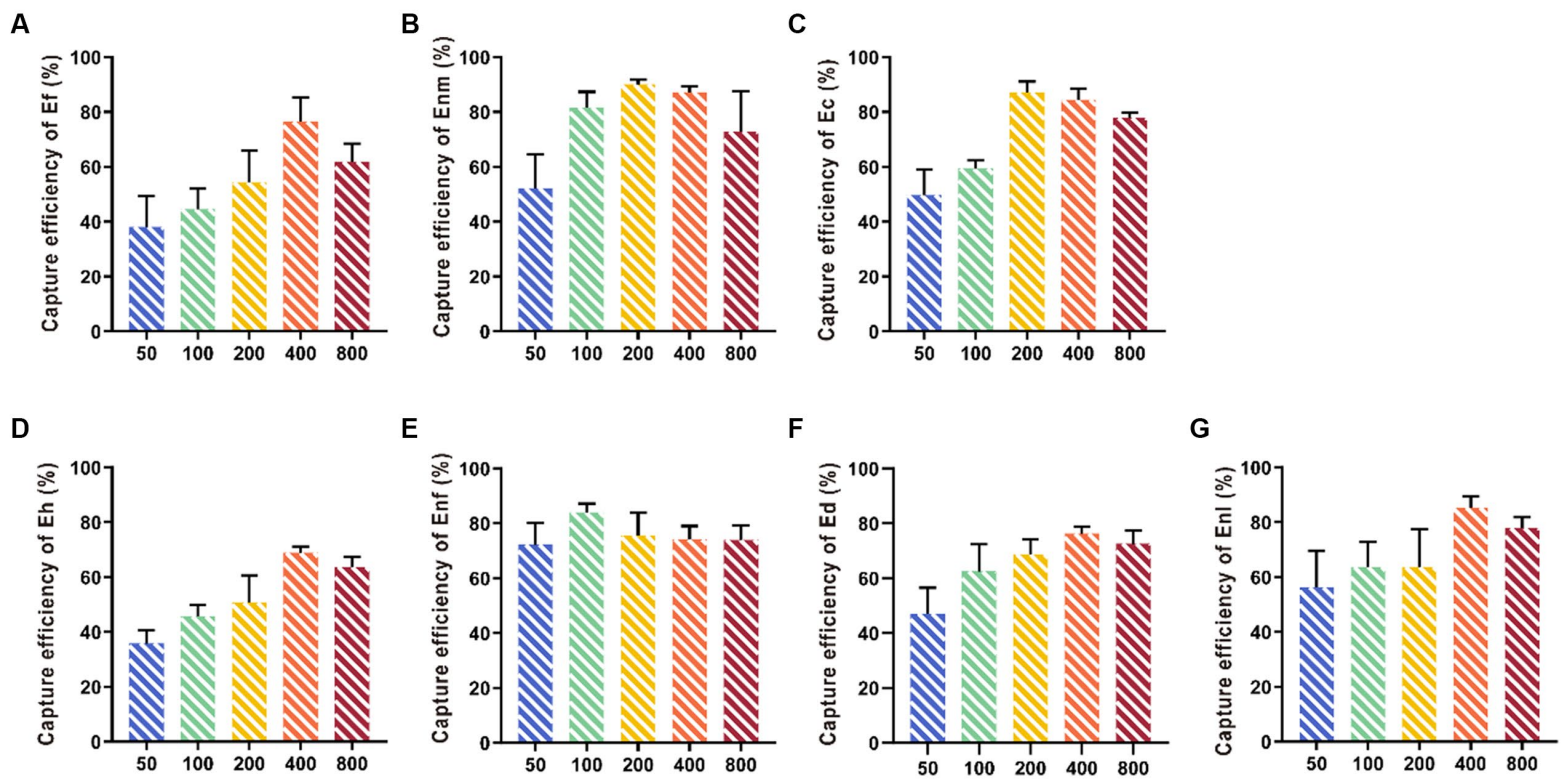
Optimization of multi-epitope anti-coupling IMBs. Various concentrations (50, 100, 200, 400, and 800  μg/mL) of multi-epitope antibody En dltD were coated on beads. The CE values of **(A-G)** Ef, Enm, Ec, Eh, Enf, Ed, and Enl by IMBs were affected by the amount of antibody.

The capture efficiency of Enm, Ec, and Enf by IMBs showed an increasing trend with antibody dosage ranging from 50 μg to 200 μg (Figure 4). When the antibody dose exceeded 200 μg, the capture efficiency began to decrease, and when the antibody dose was between 50 and 400 μg, the capture efficiency of Ef, Ed, Enl, and Eh gradually increased. The CE values of Enm, Ec, and Enf were 88.36, 87.10, and 84%, respectively, when the optimal amount of antibody added was 200 μg, and the optimal amount of antibody added to capture Ef, Ed, Enl, and Eh was 400 μg, and the CE values were 73.74, 76.83, 82.1, and 72.18%.

#### 3.3.2 Optimization of IMB dosage

**Figure 5:**
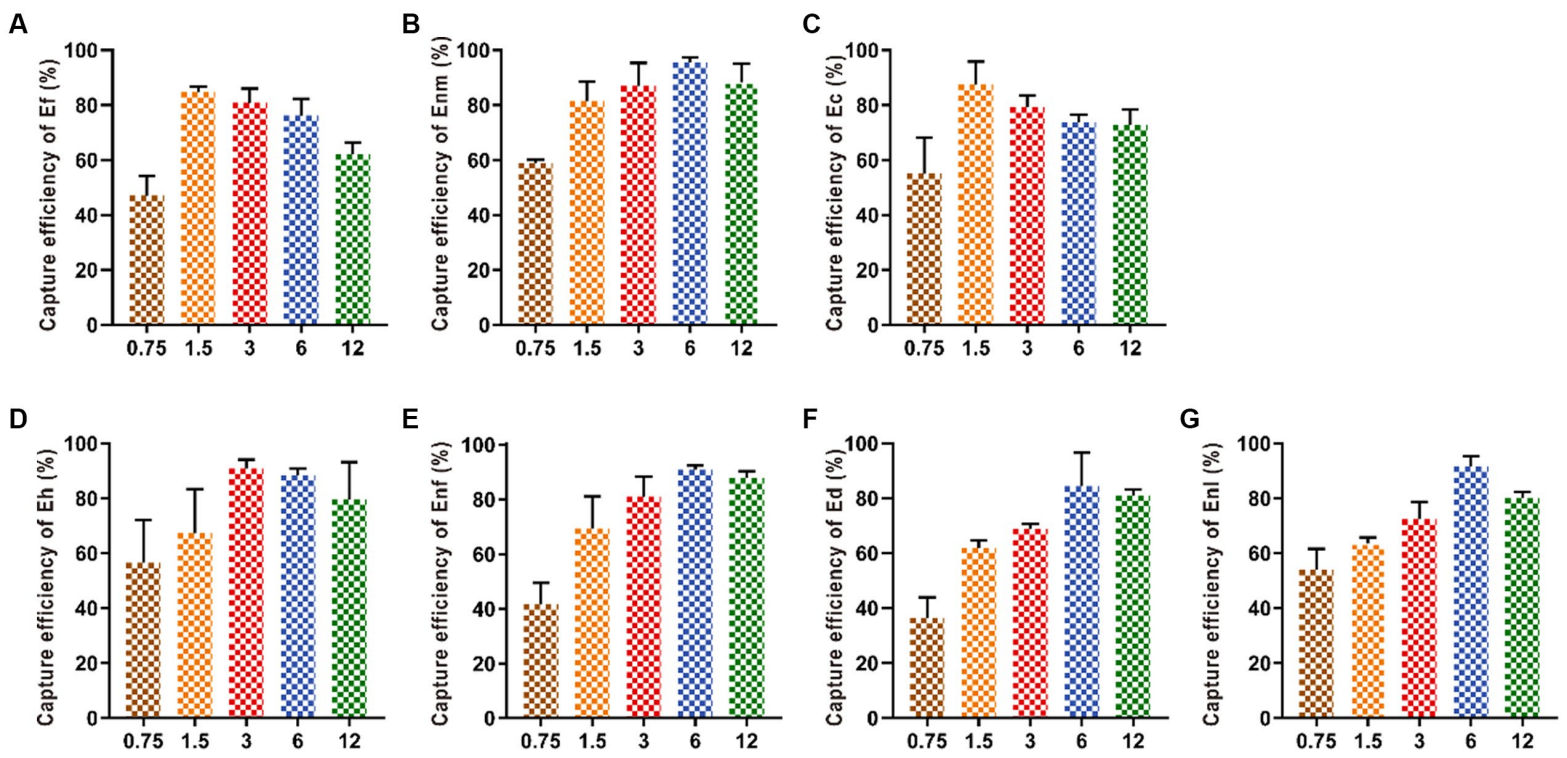
Optimization of the amount of IMBs added. Various amounts (0.75, 1.5, 3, 6, and 12 mg) of IMBs were added in the bacterial suspension. The CE values of **(A-G)** Ef, Enm, Ec, Eh, Enf, Ed and Enl were affected by the amount of IMBs added.

Increasing the amount of added IMBs from 0.75 mg to 1.5 mg increased the capture efficiency of Ef and Ec by IMBs up to 80.66 and 87.5%, respectively. When the addition amount of MBs exceeded 1.5 mg, the capture efficiency began to decline. When the amount of MBs was 3 mg, the capture efficiency of Eh reached the highest value of 84.42%. With the addition of MBs increased from 0.75 mg to 6 mg, the capture efficiencies of Ed, Enm, Enl, and Enf gradually increased and finally leveled off (Figure 5).

#### 3.3.3 Optimization of immune reaction time for IMB

**Figure 6:**
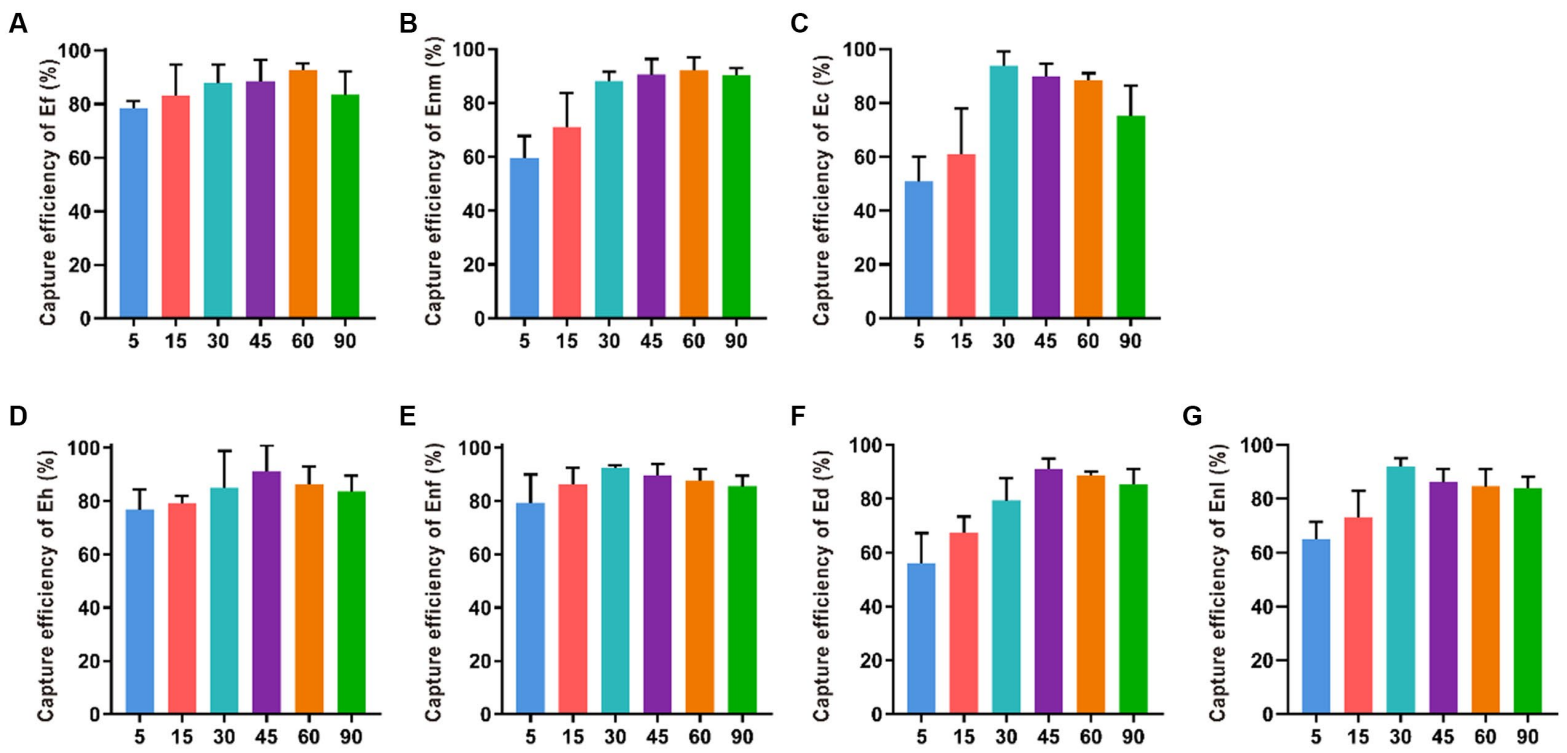
Optimization of reaction time between IMBs with 7 enterococci. The immune response time was set to 5, 15, 30, 45, 60 and 90 min to investigate their effect on the CE of **(A-G)** Ef, Enm, Ec, Eh, Enf, Ed and Enl.

The CE values of Ec, Enl, and Enf gradually increased within 5–30 min of immune reaction were 93.87, 91.96, and 92.30%, indicating that the longer the reaction time between the bacterial solution and the immune magnetic beads, the higher the capture rate (Figure 6). However, when the immune response time was more than 45 min, the capture rate showed a downward trend. When the immune response time was 45 min, the capture rate of Ed and Eh was the highest, reaching 92.16 and 87.94%. At 60 min, Ef and Enm had the highest capture efficiencies of 93.65 and 93.79%, respectively.

#### 3.3.4 Optimization of the immunomagnetic separation time

**Figure 7:**
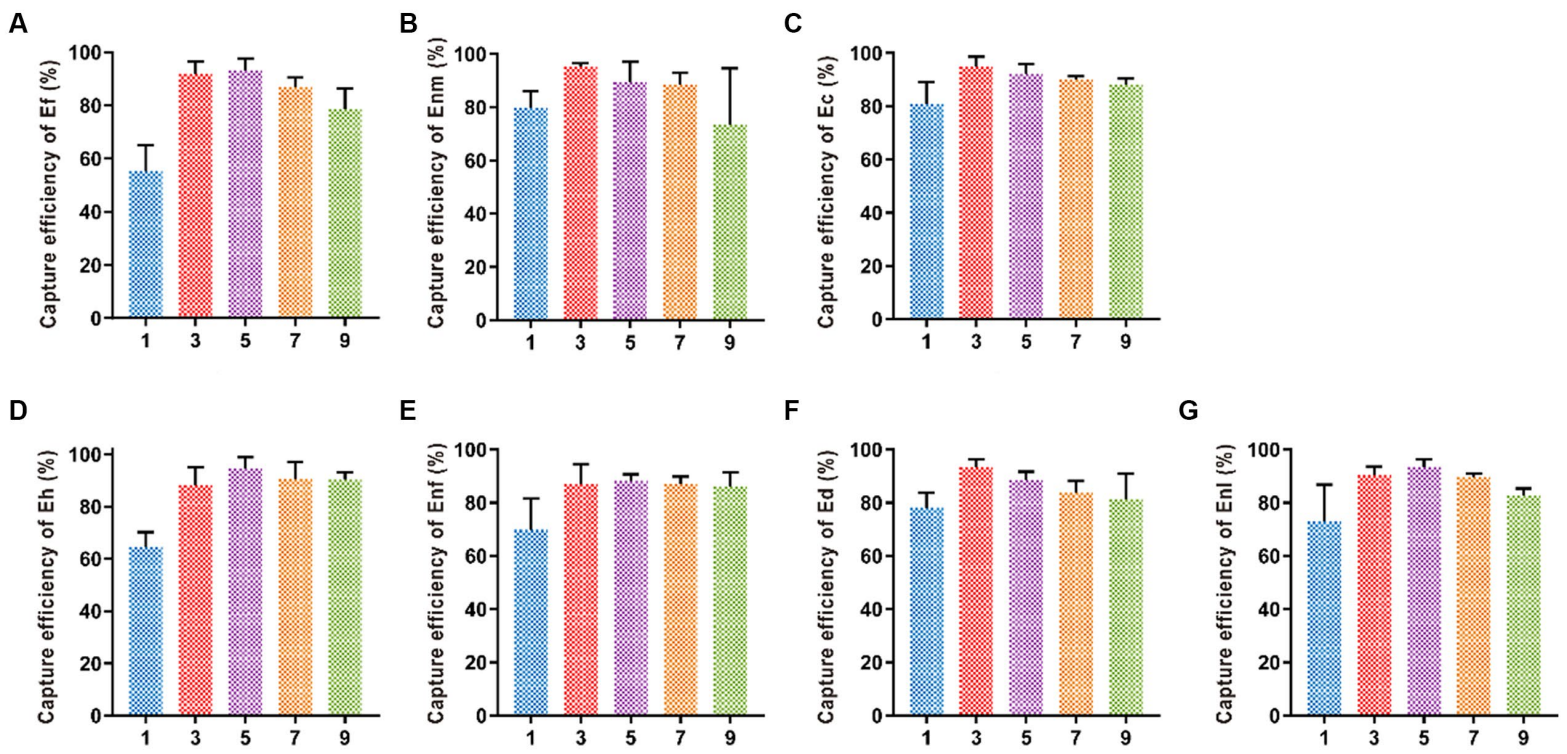
Optimization of immunomagnetic separation time. The immunomagnetic separation times were set to 1, 3, 5, 7, and 9 min to investigate the time on CE of **(A-G)** Ef, Enm, Ec, Eh, Enf, Ed, and Enl.

Five different time periods of 1, 3, 5, 7, and 9 min were selected for immunomagnetic separation, and the best magnetic separation time of Enl and Eh was 5 min with capture efficiencies of 92.09 and 87.99%, respectively (Figure 7). When the capture efficiency of Ef, Ec, Ed, Enm, and Enf reached the highest, the magnetic separation time was 3 min, and the capture efficiency decreased after 3 min, indicating the best magnetic separation time was 3 min. The capture efficiencies of Ef, Ec, Ed, Enm, and Enf were 91.9, 94.98, 93.07, 93.19, and 93.57%, respectively.

#### 3.3.5 Optimization of the buffer pH

**Figure 8:**
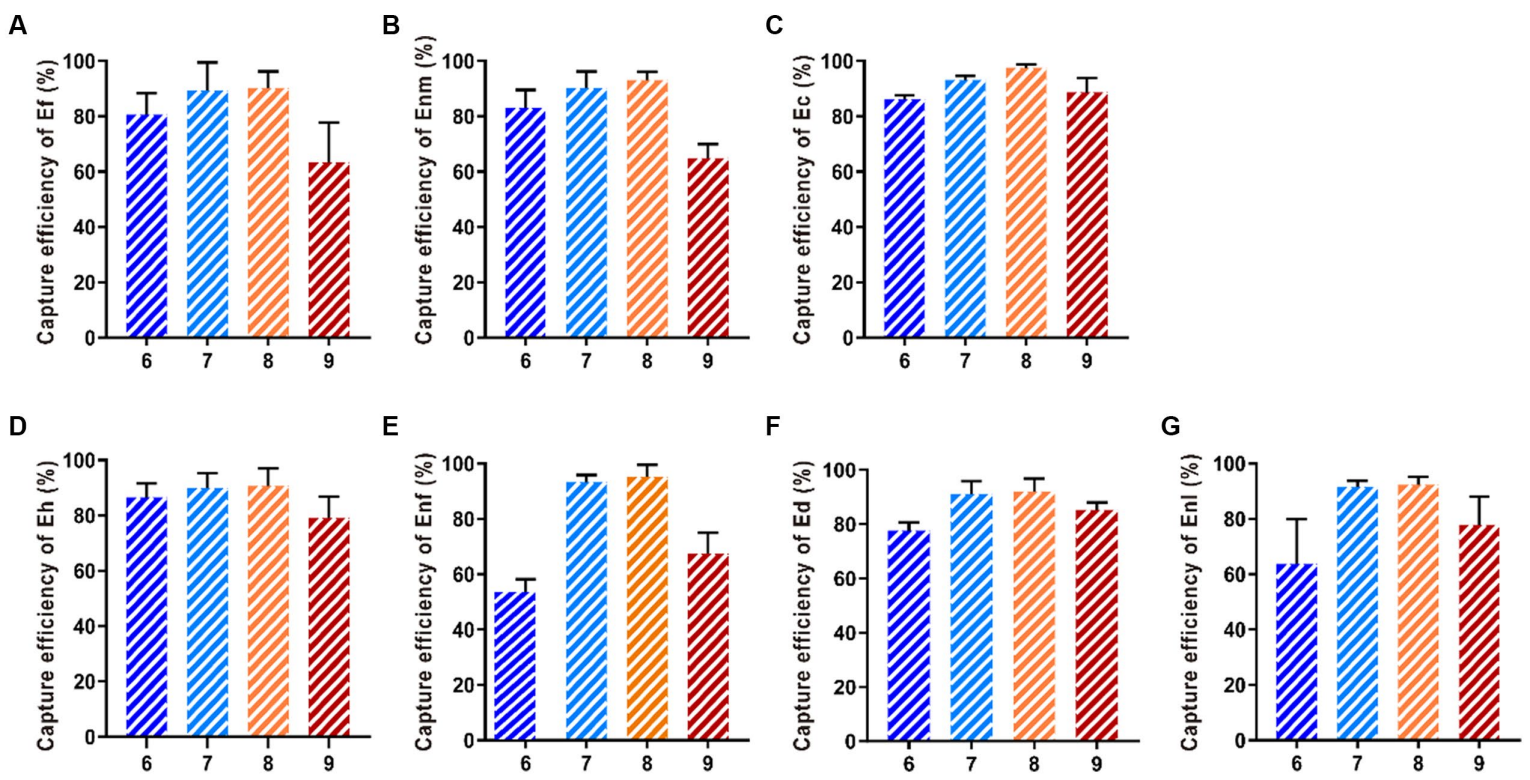
Optimization of buffer pH. Buffer pH values of 6, 7, 8, and 9 were selected to investigate the effect of buffer pH on the CE of **(A-G)** Ef, Enm, Ec, Eh, Enf, Ed, and Enl.

The capture efficiency of Enf in PBS with pH 7.0 was 93.68%. The capture efficiencies of Ef, Enl, Ec, Eh, Ed, and Enm in PBS with pH 8.0 were 95.07, 92.31, 97.58, 90.63, 92.07, and 93.06%, respectively. In PBS with pH 9.0, the CE is in a declining state (Figure 8).

#### 3.3.6 IMBs performance analysis and microstructure observation

**Figure 9:**
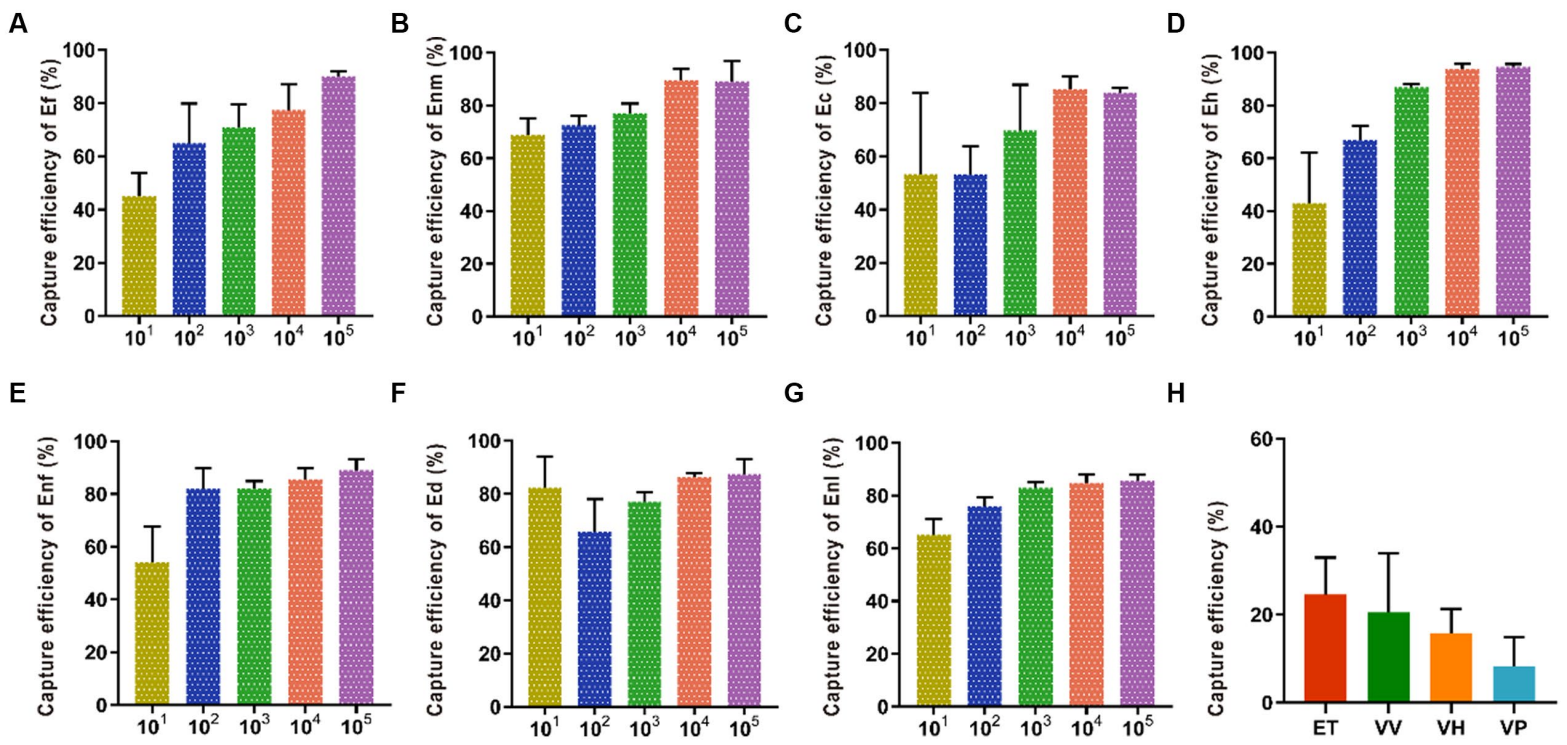
IMB sensitivity and specificity detection. The range of bacterial concentration in the IMBs sensitivity test was selected from 101 to 105 CFU/mL were selected to investigate the bacterial concentration on CE of **(A-G)** Enf, Ef, Enl, Ec, Eh, Ed, Enm and **(H)** Vp, Vv, Vh, and Et were selected to detect the specificity of IMBs.

Under the optimum conditions, according to 3 M petrifilm plates test results (Supplementary Figure S2), the CE was over 70%, going as high as 97.58%, except for 10^1^ CFU/mL, when the IMB was used to capture Ec, Ef, Enl, Eh, Enf, Enm, and Ed in the range of 10^1^–10^5^ CFU/mL. By contrast, the capture efficiencies of Vp, Vv, Vh, and Et were lower than 24.60% on average (Figure 9).

**Figure 10:**
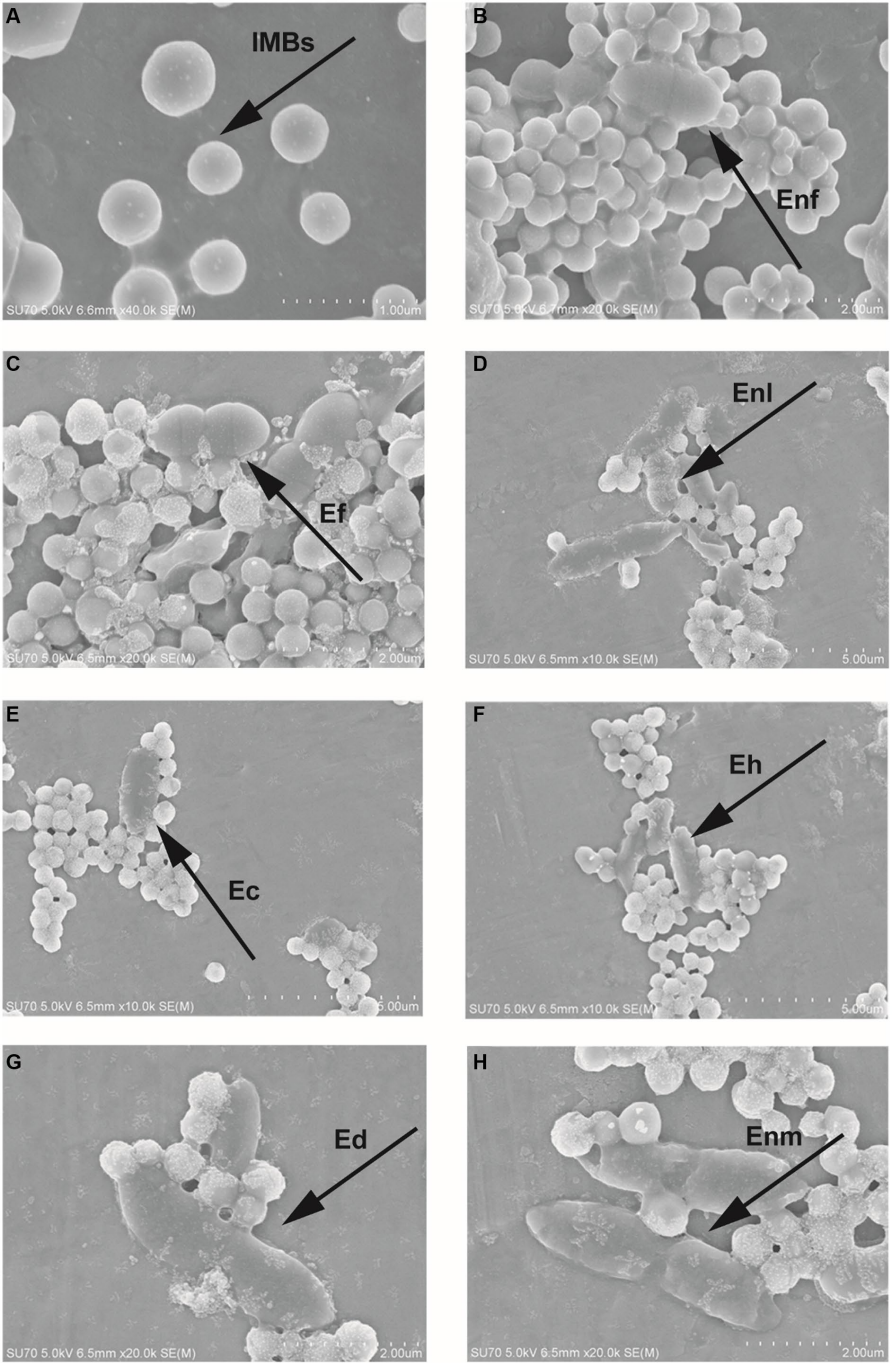
The result of SEM. **(A)** IMB conjugated antibody. The results show the microstructure of **(B-H)** Enf, Ef, Enl, Ec, Eh, Ed and Enm connected with magnetic beads. The round ones are IMBs, and the oval structures are enterococci.

SEM results showed that most of the nano-magnetic beads were regular and round with good dispersion and uniform size (Figure 10A). The figure shows that IMBs are linked to various enterococci by surface antibodies, and each bacterium may be bound by several magnetic beads, thus forming a magnetic beads-bacteria complex, which appears to be aggregated under the electron microscope. These images in Figures 10B–G show that seven enterococci, namely, Enf, Ef, Enl, Ec, Eh, Ed, and Enm, bind to antibodies on the surface of IMBs, and a large number of magnetic beads are bound to the surface of the bacteria.

### 3.4 Determination of the labeling conditions of colloidal gold

The prepared colloidal gold solution is clear and transparent, red, and has no suspended material on the surface and no accumulated particles at the bottom (Supplementary Figure S3A). The quality of colloidal gold was analyzed by transmission electron microscope and using a UV spectrum scanner. TEM results showed that the average particle size of colloidal gold prepared was about 24 nm with uniform dispersion and regular shape (Supplementary Figure S3B). The maximum absorption peak (*λ*_max_) was 524 nm in the range of 400–800 nm as determined using the UV spectrum scanner, and only one peak with smooth waveform and narrow peak shape (Supplementary Figure S3C) was observed, indicating that the colloidal gold solution prepared was of good quality.

**Figure 11:**
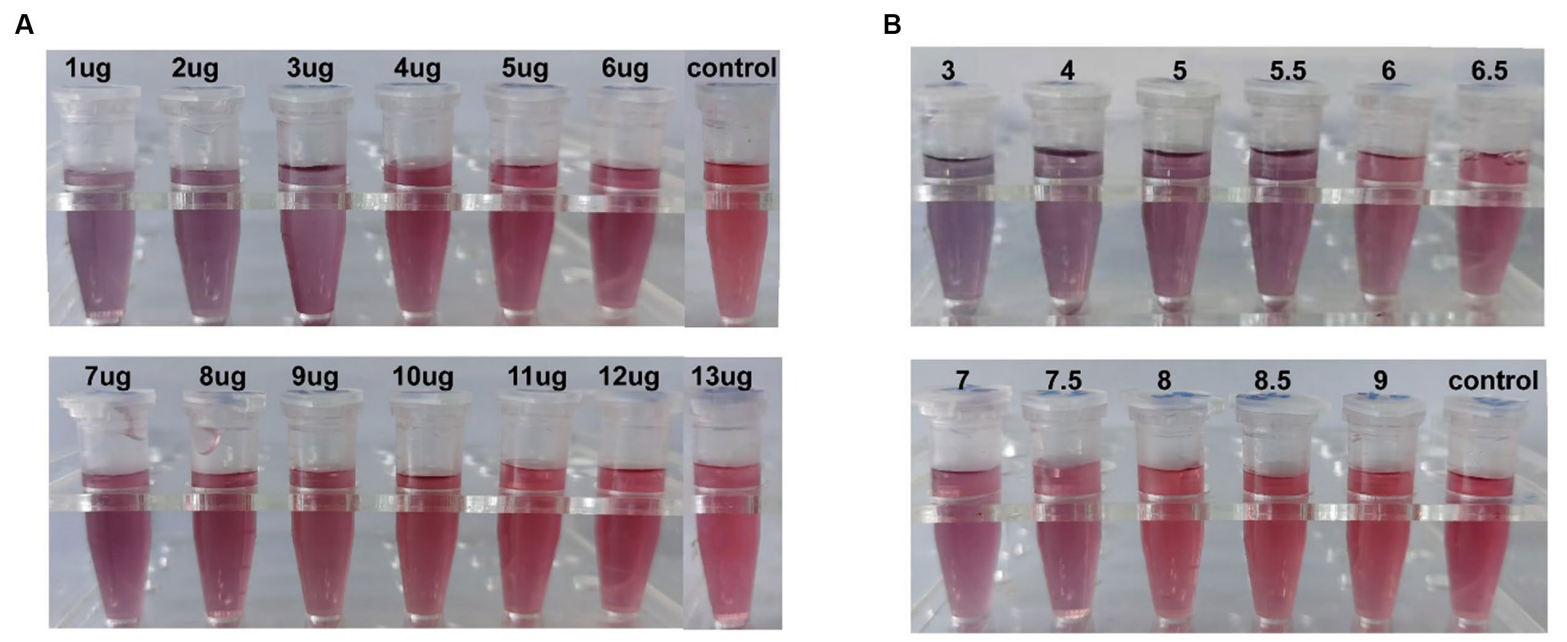
Optimization of colloidal gold labeling conditions. **(A)** The gold color of the colloid changes when adding different amounts of antibody. **(B)** pH is different at the same time the color change of colloidal gold. The control group was not added with the antibody.

According to Mey’s stability test, when the amount of artificial multi-epitope antibody labeling was 12 μL/mL, the color of colloidal gold tended to be stable (Figure 11A). Therefore, the optimal labeling amount was 14.4 μL/mL. When the pH was between 3 and 7.5, the colloidal gold solution became gray or light in color, and at pH 8.0, the colloidal gold solution maintained a red color (Figure 11B). Consequently, pH 8.0 was selected as the optimal marker pH value for multi-epitope antibody immunocolloidal gold.

### 3.5 Specificity of ICTS

**Figure 12:**
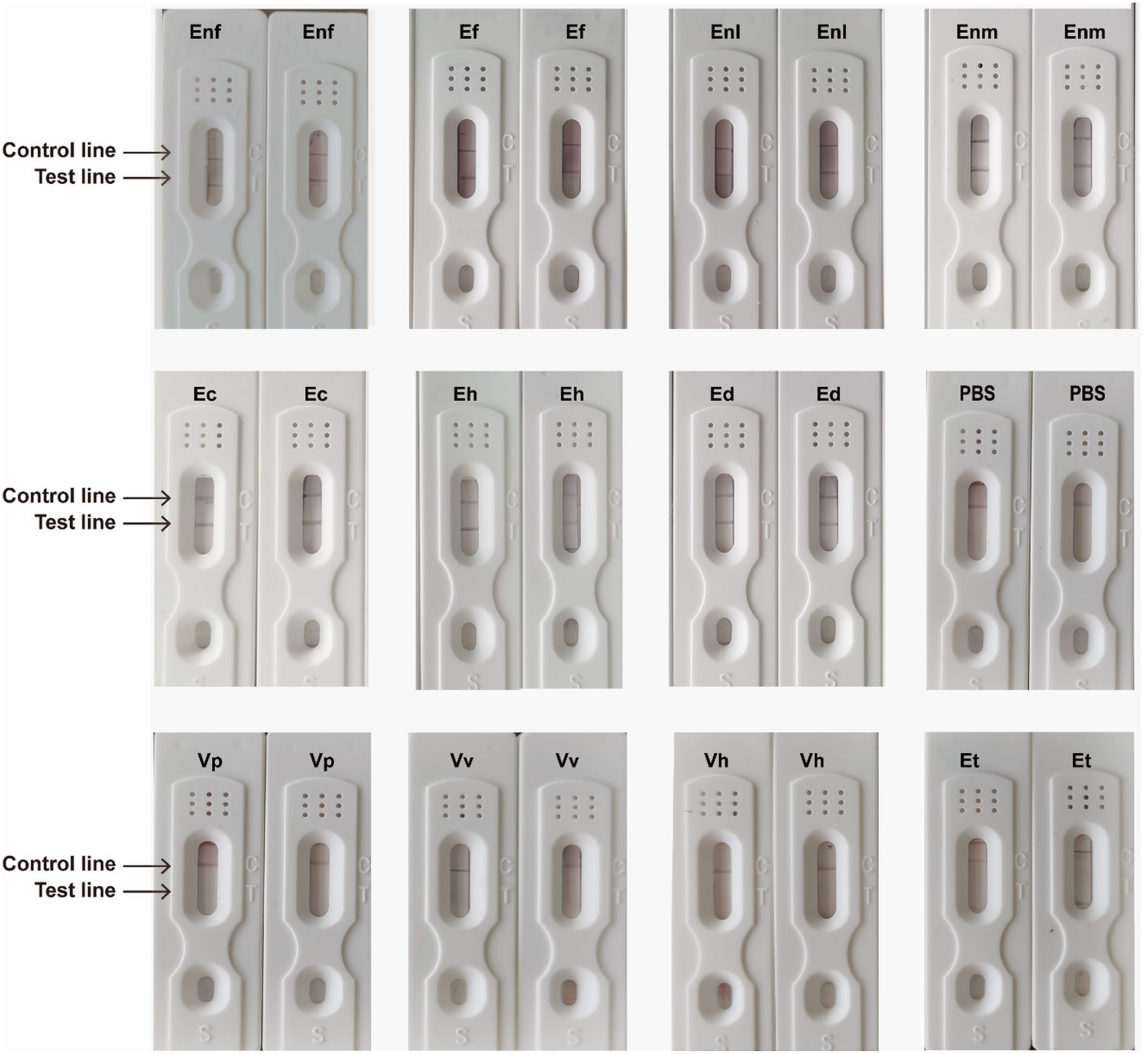
Specificity testing of the ICTS. The positive specimens included Enf, Ef, Enl, Ec, Eh, Ed, and Enm, and negative samples from Vp, Vv, Vh and Et and PBS were simultaneously tested by this strip test.

Kim et al. (2023) showed that the abundance of Vibrio was higher in the samples from the marine bathing beach, and we selected *V. parahemolyticus*, *V. vulnifcus*, *V. harveyi*, *V. anguillarum*, and *E. tarda* to detect the specificity of the developed method for enterococci bacteria. After the specificity of ICTS was tested by enterococci and Vp, Vv, Vh, and Et, the ICTS prepared with artificial multi-epitope antibody showed that seven kinds of enterococci were positive. PBS was used as the blank control, and the specificity detection results of Vp, Vv, Vh, and Et were all negative, indicating that the test strip has good specificity (Figure 12).

### 3.6 Detection evaluation of IMS-PCR

The direct use of the ICTS method can only detect enterococci higher than 10^2^ CFU/mL, and the limit of detection of enterococci by IMS-ICTS method is 10^1^ CFU/mL (Supplementary Table S1). In comparison with the ICTS method, the sensitivity of the detection of enterococci by IMS-ICTS increased by 10 times.

## 4 Discussion

Epitope chemical group with a specific structure that exists on the surface of antigens can be specifically recognized by antibodies (Agarwal et al., 2022). Epitopes are target structures that are recognized by immune cells. A single antigenic molecule may have one or more distinct epitopes, and each epitope is specific for only one antigen. At present, the construction of multi-antigen epitopes is mainly used in vaccine design to combat diseases, including tumors and various pathogenic bacteria (Al-Megrin et al., 2022; Saha et al., 2022). Mahdevar et al. (2022) designed a novel multi-epitope vaccine against breast cancer, which comprised of the most immunodominant epitope of the BORIS cancer-testis antigen by using immune-informatics approach. Albutti (2022) designed a multi-epitope vaccine structure that targets highly specific antigenic epitopes of the four target proteins of Rift Valley fever (RVF) and evaluated its potential immune effect, thus providing a new direction for future vaccines against RVF virus. Accordingly, the use of immune-informatics methods for the design of multi-epitope vaccines with high practical value has been become an attractive means of vaccine research, thus providing support for the prevention of many diseases in the future (Molina-Franky et al., 2020; Saha et al., 2022).

Notably, the multi-epitope antibodies prepared in this experiment are polyclonal antibodies. Polyclonal antibodies are also widely used to connect with magnetic beads for the separation of target proteins (Zhang et al., 2020; Schuster et al., 2022). Although monoclonal antibodies have high specificity, they can only specifically recognize a single epitope, and the production process is complicated. In comparison with monoclonal antibodies, polyclonal antibodies have the advantages of easy large-scale preparation and recognition of multiple epitopes on antigens to better play the advantages of multiple epitopes (Dean Goldriig et al., 1989; Schmidt et al., 2021). This experiment made full use of the advantages of polyclonal antibodies, and multi-epitope polyclonal antibodies were prepared for the trace and rapid detection of water quality in environmental water samples and recreational water. The whole process could simultaneously complete trace detection of seven kinds of pathogenic enterococci, including Enf, Ef, Enl, Ec, Eh, Ed, and Enm. The detection process does not take more than 1 h.

At present, IMBs can be divided into amino magnetic, carboxyl magnetic, and silicon magnetic beads according to different surface groups. Carboxyl-coated magnetic beads are widely used in pathogen detection (Wang L. et al., 2022). Chai and Bi (2022) performed enrichment and capture of *E. coli* with anti-*E. coli*-modified carboxyl magnetic beads and detected *E. coli* from complex matrices. Wang Z. et al. (2022) pretreated *E. coli* with carboxy group-coated IMBs and established a method for the rapid detection of *E. coli* O157 cells in fresh strawberry and lettuce. In comparison with micro-magnetic beads, nano-magnetic beads have the advantages of larger specific surface area and better suspension stability, resulting in lower detection limit and easier separation (Brandão et al., 2015). Li et al. (2019) detected *Salmonella* in milk and chicken by immunomagnetic separation. Although the applied micron magnetic beads can detect bacteria, the CE is less than 75%, and the detection time is long. The IMBs used in the present experiment are nano-magnetic beads with CE of up to 90%, and whole detection process was completed within 1 h. This finding can be attributed to the small and uniform size of nano-magnetic beads, which are greatly affected by Brownian motion, and the probability of contacting antigen in solution increased. Furthermore, considering that PCR is the most commonly used molecular detection method in this field, the specificity of the IMBs can be confirmed by verifying the products of magnetic bead enrichment with specific primers, and the results showed that the IMBs could specifically recognize the target strain. ICTS can be used to detect pathogens easily and quickly, and the colloidal gold particle size for the detection of pathogens is usually 20–25 nm (Sun et al., 2014). TEM images of the selected colloidal gold showed that the colloidal gold was uniform in size and evenly dispersed with a diameter of about 24 nm (Supplementary Figure S3B), and based on the UV spectrum scanner results, the maximum absorption peak was 524 nm with only one peak and a smooth wave (Supplementary Figure S3C), indicating good quality. Accurate ICTS results were obtained by performing a series of optimizations, including the amount of labeled multi-epitope antibodies used (Figure 11A), the pH of the colloidal gold fluid (Figure 11B), and goat anti-mouse IgG. After optimization, the results can be observed within 10 min (Figure 12). During the experiment, the carboxyl groups on the surface of magnetic beads were first activated by N-(3-dimethylaminopropyl)-N′-ethyl carbondiimide hydrochloride (EDC) solution. Then, the antibodies (multi-epitope antibodies in this article) were attached to the activated magnetic bead surface, and then BSA was added to block nonspecific adsorption sites on the IMB surface. MST technology (Zhang et al., 2019) was used to verify the affinity of multi-epitope antibodies to pathogenic bacteria (Figure 3), IMS and ICTS test the sensitivity of the antibody The multi-epitope antibody had high specificity for the target bacteria in MST, IMS, and ICTS and can realize the rapid detection of enterococci. The detection minimum was 10^1^ CFU/mL, and incompatibility was observed for Vp, Vv, Vh, and Et (Figure 9). Therefore, the multi-epitope antigens screened using this method are highly conserved and have research value. We will investigate the application in bathing beach samples in the future work.

## 5 Conclusion

In conclusion, a multi-epitope antigen was generated against seven major pathogenic enterococci, and polyclonal antibodies were obtained by animal immunization. IMS-ICTS was used to complete the enrichment and field detection of various enterococci. Results showed that the antibody had good specificity and sensitivity and is suitable for the rapid detection of target bacteria by IMBs, ICTS, and MST. In this investigation, the whole detection process was completed within 1 h, including sample processing time and detection time, which do not require complex operations and sophisticated laboratory equipment. It achieves the purpose of water quality monitoring in bathing beach and solves the problems of complex substrate composition and difficult separation of target bacteria.

## Data availability statement

The original contributions presented in the study are included in the article/Supplementary material, further inquiries can be directed to the corresponding authors.

## Author contributions

YL: Investigation, Methodology, Visualization, Writing – original draft. ZiyanW: Writing – review & editing. ZeW: Software, Writing – original draft. JZ: Data curation, Funding acquisition, Writing – review & editing. JH: Writing – review & editing. CL: Methodology, Visualization, Writing – review & editing. BL: Methodology, Writing – review & editing. RY: Conceptualization, Writing – review & editing. XSun: Data curation, Resources, Writing – original draft. ZZ: Funding acquisition, Supervision, Writing – review & editing. RW: Supervision, Writing – review & editing. XSu: Supervision, Writing – review & editing.

## Footnotes

^1^ https://www.ncbi.nlm.nih.gov

^2^ https://www.iedb.org

^3^ https://swissmodel.expasy.org/
