# Supplementary material for: Rapid and simultaneous multiepitope antigen-based detection of Enterococcus by microscale thermophoresis and immunomagnetic separation
Source: Front Microbiol. 2024 Jan 23;15:1341451. doi: 10.3389/fmicb.2024.1341451 (PMC10844561; doi:10.3389/fmicb.2024.1341451)
Supplement: Supplementary file 1 [file Data_Sheet_1.docx]

**Rapid and simultaneous multi-epitope antigen-based** **detection of *Enterococcus* by microscale thermophoresis and immunomagnetic separation**

Yan Liu^1,2^, Ziyan Wang^1,2^, Ze Wang^1,2^, Jun Zhou^1,2^, Jiaojiao Han^1,2^, Chenyang Lu ^1,2^, Bing Liu^3^, Rongxian Yu^3^, Xiaoling Sun^3^, Zhen Zhang^1,2*^, Rixin Wang^2*^, Xiurong Su^1,2*^

1. *State Key Laboratory for Quality and Safety of Argo-products, Ningbo University, Ningbo* 315201, *China*
2. *School of Marine Science, Ningbo University, Ningbo* 315832, *China*
3. *Vigor Health Products Co., Ltd, Shenzhen* 518118, *China*

Corresponding author:

Zhen Zhang, Rixin Wang, Xiurong Su

169 Qixing South Road,

Ningbo University,

Ningbo,

Zhejiang Province 315832, P. R. China

Email: [zhangzhen@nbu.edu.cn](mailto:zhangzhen@nbu.edu.cn), [wrx_zjou@163.com](mailto:wrx_zjou@163.com), suxiurong_public@163.com

**Supplementary materials and methods**

**Specific test**

Approximately 3 mg IMBs was added to 1 mL of Vp, Vv, Vh, Va, and Et, and the concentration of bacteria ranged from 10^1^ to 10^5^ CFU/mL. All centrifuge tubes were placed on a tube rotator for 45 min at 37 ℃. After magnetic separation, and the sample was washed thrice with PBS, re-suspended with PBS, and inoculated on 3M petrifilm plates with three parallel treatments. The samples were cultured at 28 °C for 1–2 days and the capture efficiency was calculated.

**Preparation of colloidal gold**

Colloidal gold was prepared by trisodium citrate reduction. For the specific synthesis, 1.0 mL of 1% (*w*/*v*) HAuCl_4_ solution was added into 99.0 mL of ultrapure water, and the solution was then heated to boiling with stirring. 0.75 mL of 2% C_6_H_5_Na_3_O_7_·2H_2_O solution was added quickly after constant heating and stirring with a change in solution color. The color of the solution changed from light yellow to black, and then to brown, and finally to red. Finally, the solution turned transparent red. The solution was boiled for 10 min, and the heat source was removed. The volume was set to 100 mL, and the gold colloidal solution was stored at 4 ℃ for several months. The cooled colloid was stored at 4 ℃ in a dark bottle.

**Determine the optimal pH for labeling**

Approximately 1 mL of colloidal gold solution was successively added to 1.5 mL EP tubes, the pH of the AuNPs were adjusted with 0.2 mol/L K_2_CO_3_. Multi-epitope antibody (L mg/mL) and 100 μL 10% NaCl solution were added. All solutions were added and left for 15 min, the color change was observed, and the best labeled pH value is the lowest pH value when the colloid gold solution color remains red.

**Determine the optimal amount of antibody for labeling**

The optimal amount of antibody for labeling was determined by visual inspection. Twelve 1.5 mL EP tubes were obtained, and each tube was added with 1 mL of colloidal gold solution. The pH of the AuNPs was adjusted with 0.2 mol/L K_2_CO_3_. Varying volumes of enterococcal multi-epitope antibody MAb and 100 μL 10% NaCl were added. The solution was mixed well and left to stand at room temperature for 2 h. The amount of antibody in the colloidal gold solution remains red, and an additional 20% is the optimal amount of antibody labeling.

**Supplementary figures and table**

**Figure S1 Results of Coomassie brilliant blue staining**. 1: Protein before IPTG induction; 2: Protein induced by IPTG; 3: Broken thallus precipitates; 4: 50 imidazole flow through the solution; 5: 70 imidazole flow through the solution; 6: Ultrafiltration tube outflow; 7: Before the enrichment; 8: After enrichment; M: Marker.

**
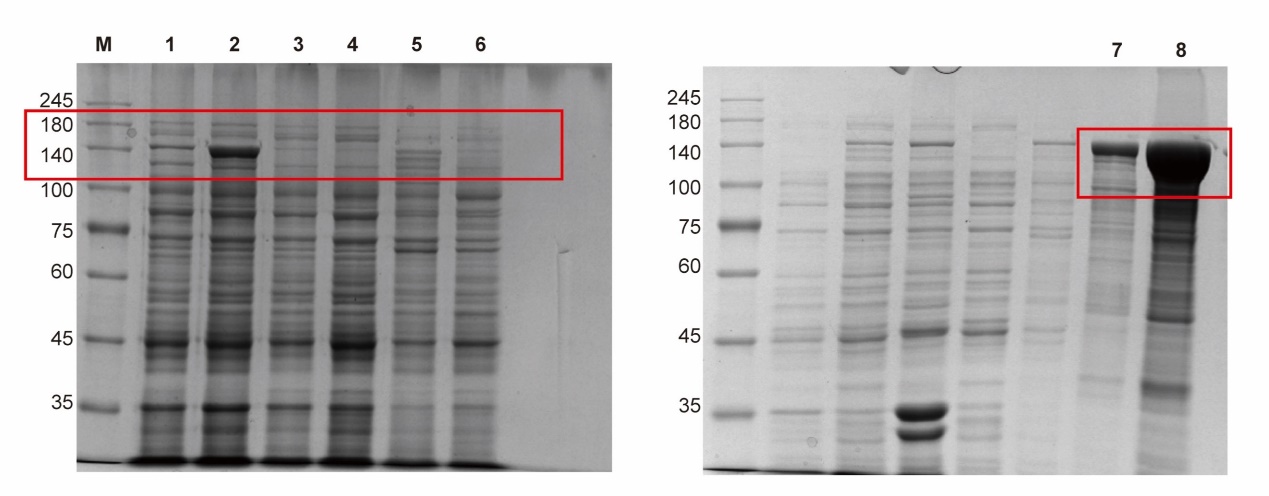
**

**Figure S2 The capture of** **non-target and target bacteria by 3M petrilm plates.** Target bacteria include *Enterococcus faecium* (Enf), *E. faecalis* (Ef), *E. lactis* (Enl), *E. casseliflavus* (Ec), *E. hirae* (Eh), *E. durans* (Ed) and *E. mundtii* (Enm); Non-target include *Vibrio parahemolyticus* (Vp), *V vulnifcus* (Vv), *V. harveyi* (Vh), *V. anguillarum* (Va), and *E. tarda* (Et).


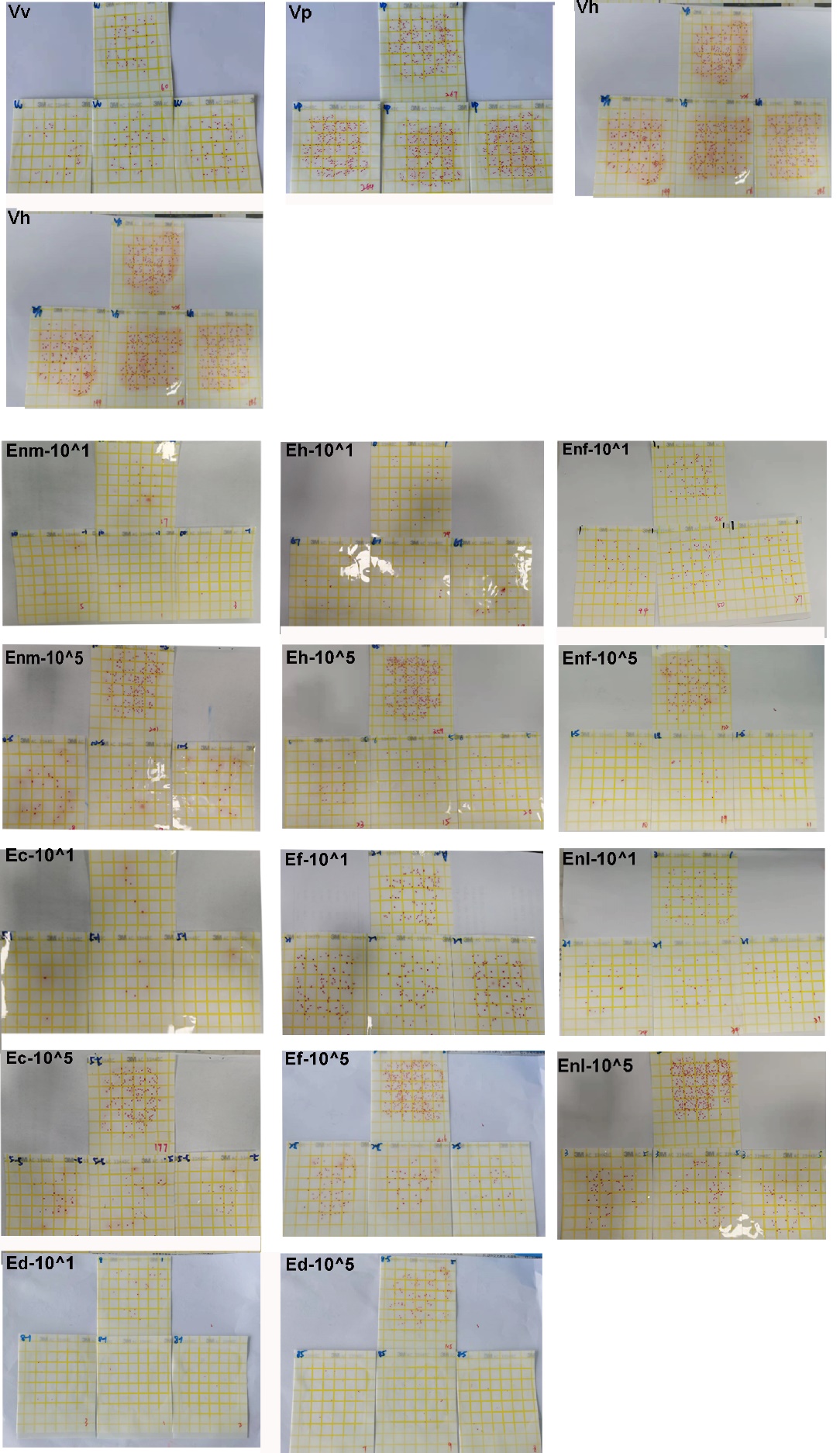


**Figure S3 Colloidal gold quality assessment results.** (a) Colloid gold solution color results, solution clarification without precipitation; (b) SEM results showed that the colloidal gold particles were uniformly dispersed without aggregation, and the particle size was uniform; (c) The maximum absorption peak λ Max =524 nm in the range of 400-800 nm was observed by UV spectrum scanner, and there was only one peak with smooth waveform and narrow peak shape. The above results indicate that the quality of colloidal gold is better.


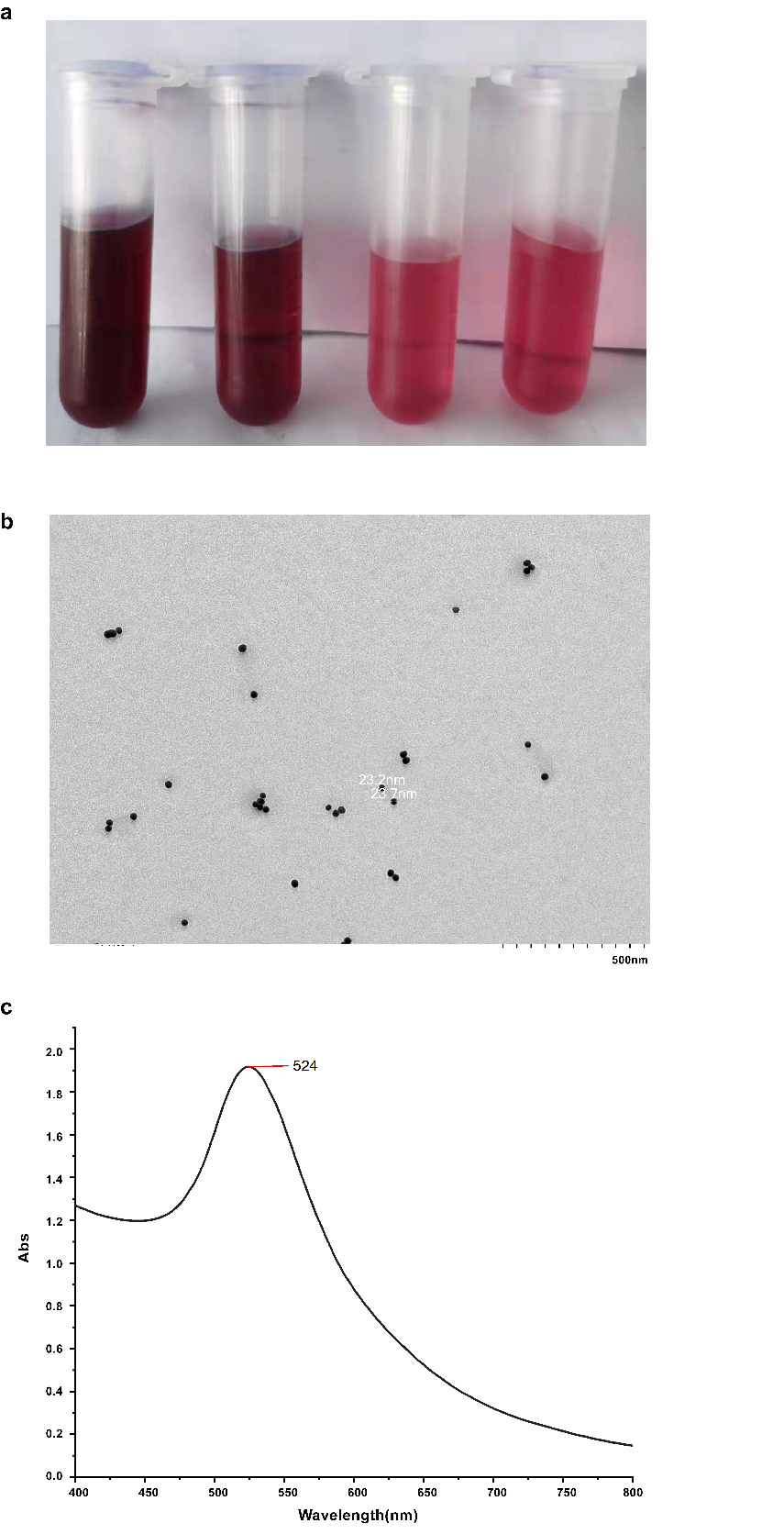


**Table S1 The results of pure culture enterococci detected by two methods were compared.**

| Bacteria | Results of colloidal gold immunochromatography | Bacterial liquid concentration /CFU·mL^-1^ | | | | |
| --- | --- | --- | --- | --- | --- | --- |
|  |  | 10^1^ | 10^2^ | 10^3^ | 10^4^ | 10^5^ |
| Enf | Not enriched | - | + | + | + | + |
|  | Enriched | + | + | + | + | + |
| Ef | Not enriched | - | + | + | + | + |
|  | Enriched | + | + | + | + | + |
| Enl | Not enriched | - | + | + | + | + |
|  | Enriched | + | + | + | + | + |
| Ec | Not enriched | - | + | + | + | + |
|  | Enriched | + | + | + | + | + |
| Eh | Not enriched | - | + | + | + | + |
|  | Enriched | + | + | + | + | + |
| Ed | Not enriched | - | + | + | + | + |
|  | Enriched | + | + | + | + | + |
| Enm | Not enriched | - | + | + | + | + |
|  | Enriched | + | + | + | + | + |

"-" means negative, "+" means positive; Not enriched means not enriched by IMBs; Enriched means enriched by IMBs.
